# May Subjective Language Complaints Predict Future Language Decline in Community-Dwelling Subjects?

**DOI:** 10.3389/fpsyg.2019.01974

**Published:** 2019-08-28

**Authors:** Carolina Maruta, Isabel Pavão Martins

**Affiliations:** ^1^Language Research Laboratory, Centro de Estudos Egas Moniz, Faculty of Medicine, University of Lisbon, Lisbon, Portugal; ^2^Católica Research Center for Psychological, Family, and Social Wellbeing, Faculty of Human Sciences, Universidade Católica Portuguesa, Lisbon, Portugal; ^3^Department of Neurology, Centro Hospitalar Lisboa Norte, Lisbon, Portugal

**Keywords:** cognitive decline, aging, verbal fluency, subjective language complaints, subjective memory complaints questionnaire

## Abstract

Subjective cognitive complaints are rather prevalent in the elderly population and are associated with an increased risk of cognitive impairment and dementia. However, the predictive role of specific types of cognitive complaints has been less systematically assessed. The aim of the present study is to examine the predictive value of language complaints for cognitive and language decline in a cohort of community-dwelling healthy older adults, followed longitudinally over a 5-year period. A total of 402 subjects were enrolled in a prospective longitudinal study on aging and cognition. Participants answered a cognitive complaints questionnaire including two questions directed to language and were classified at baseline as having “*Language Complaints*” (LC) or “*No Language Complaints*” (NLC). They also performed a neuropsychological assessment tackling attention/processing speed, memory, executive functioning, and language at baseline. From these, 275 (68.4%) participated in a follow-up evaluation 4.9 (±0.6) years later. At re-evaluation, subjects had a mean age of 70.4 (±8.3) years, 7.5 (±4.4) years of education, and 63.3% were female. Multivariate linear regression analysis was used to investigate whether language complaints at baseline predicted poorer language performance at follow-up or increased the risk of cognitive decline, with correction for sex, depressive symptoms, living status, baseline performance, and composite memory and executive performance. Results indicated that LC subjects had significantly worse performances than NLC subjects on semantic fluency 5 years later, but with a similar rate of decline overtime that was not associated with a follow-up outcome of cognitive decline/dementia. Language difficulties may represent a specific type of age-related cognitive complaints. Longer follow-ups are necessary to understand if they are associated with an increased risk of language or cognitive decline.

## Introduction

Subjective cognitive complaints (SCCs) are prevalent in the elderly populations (Slavin et al., 2010; van Harten et al., 2018). They are usually associated with a wide number of age-related conditions including hearing and vision impairments, cerebrovascular disease, and poorer perceived health (Pedro et al., 2016) and have been considered a risk factor for future cognitive decline and dementia (Abner et al., 2015).

Subjective language complaints (SLCs) are frequently reported by elderly subjects, who typically complain of difficulty in remembering proper names and word-finding problems. Cross-sectional studies indicate that SLC are associated with worse objective cognitive performance in healthy, community-dwelling subjects (Martins et al., 2012a,b) and show a pattern of increased frequency in preclinical Alzheimer’s disease (AD) when compared to controls (Valech et al., 2018). In addition, the scoring on language items in subjective complaints questionnaires has been associated with a *β* deposition (La Joie et al., 2016), which further strengths the connection between the specificity of complaints and AD.

The role of SCC in the prediction of future cognitive decline has been a matter of debate. While several studies support the relationship between SCC and poorer cognitive performance (Brailean et al., 2018) and the development of mild cognitive impairment (MCI) and AD (Jessen, 2014; van Harten et al., 2018), others have failed to establish an unequivocal association between subjective complaints and objective cognitive performance (Edmonds et al., 2014). This lack of consensus may reflect, according to some authors, that not all types of cognitive complaints might have the same clinical relevance (Ávila-Villanueva et al., 2016). They may also correspond to the dysfunction of different cognitive processes. However, the predictive role of other types of SCC beyond memory, SLC in particular, has been less systematically assessed.

The aim of the present study is to examine the predictive value of language complaints for cognitive and language decline in a cohort of community-dwelling healthy older adults, followed longitudinally over a 5-year period. We hypothesize that (1) having significant language complaints at baseline is associated with increased risk of cognitive/language decline at follow-up; (2) subjects with language complaints show significantly more decline over time on language measures when compared to subjects without complaints; and (3) having language complaints is associated with a diagnosis of cognitive decline (cognitive impairment or dementia) at follow-up.

## Materials and Methods

### Participants and Study Design

Prospective longitudinal observational cohort study on aging and cognition of 402 community-dwelling adult volunteer individuals, aged ≥50 years, attending primary care centers in the community. Subjects were recruited *via* their general practitioner if (1) Portuguese was their native language; (2) they were aged 50 years old or more; (3) they presented normal education-adjusted score on the MMSE (Folstein et al., 1975); and (4) they had autonomy for instrumental daily living activities. Exclusion criteria included the presence of any neurological, psychiatric, or medical untreated conditions able to interfere with cognition. Further details of the study design and baseline evaluation have already been published (Martins et al., 2012a,b). Briefly, participants undertook two comprehensive cognitive evaluations, one at baseline and the second after a minimum interval of 3.5-year interval. Data regarding marital, cohabitation, and professional status, vascular risk factors, co-morbidities, medication, cognitive complaints, and depressive symptoms were collected on both periods.

A total of 127 subjects previously enrolled in the baseline evaluation were lost to follow-up due to several reasons (unknown current contact/mailing address; absence of updated clinical information obtained from the local health center; and currently living outside Lisbon Metropolitan region) (Martins et al., 2018). This has led to a final cohort of 275 subjects that participated in the follow-up part of the study 4.9 (±0.6) years later on average.

The study was carried out in accordance with the recommendations of the Ethics Committee of the Lisbon Medical Academic Center with written informed consent from all subjects. All subjects gave written informed consent in accordance with the Declaration of Helsinki. The protocol was approved by the Ethics Committees of the Lisbon Medical Academic Center, Portuguese Health Authority, and local primary care centers.

### Measures

Neuropsychological evaluations were performed by fully licensed neuropsychologists. It comprised the MMSE (Folstein et al., 1975), tests of episodic [Verbal-Paired Associates, Immediate and Delayed Logical Memory, and Visual Reproduction from WMS-III (Wechsler, 1997b) and the nine-item version of the California Verbal Learning Test (Libon et al., 1996)], and semantic memory [Vocabulary subtest from WASI (Wechsler, 1999)], attention/processing speed (symbol search, WAIS-III; Wechsler, 1997a), and executive functions [Trail Making Test (A and B; Reitan, 1958) and Stroop Test (Stroop, 1935; Golden, 1978) and Digit Span Backwards (WAIS-III; Wechsler, 1997a)]. Language skills were assessed by semantic (food and animals) and phonemic verbal fluency. Individual scores were converted to age and education adjusted *z*-scores according to the existing norms (Guerreiro, 1998; Cavaco et al., 2013). A memory and executive composite score were calculated, resulting from the average standard scores obtained on two executive tests (TMT-A and B) and two episodic memory tests (Logical Memory and Verbal-Paired Associates) [(Σ*Z*_TMTA_ + *Z*_TMTB_)/2] and Memory [(Σ*Z*_Logical Memory_ + *Z*_Verbal Paired Associates_)/2], respectively.

In addition, the 15-item Geriatric Depression Scale (Yesavage et al., 1982) and the Instrumental Activities of Daily Living Scale (Lawton and Brody, 1969) were also applied.

### Language Complaints

At baseline, all participants completed a measure of subjective memory complaints, the Subjective Memory Complaints Questionnaire (SMCQ; Schmand et al., 1996). In this self-report instrument, participants are required to answer 10 individual items concerning difficulties in daily memory tasks, with total scores ranging from 0 (absence of complaints) to 21 (maximal complaints score). For the purpose of the present study, we were interested in those items tackling domains more commonly associated to language namely items 3 (directed to difficulties in proper name retrieval—“*Do you ever forget names of family member or friends”*) and 6 (referred to word finding difficulties—“*Do you ever have difficulties in finding particular words?*”). For item 3, scores range from 0 – *No* to 3 – *Yes, with problems*, whereas item 6 presents is scores 0 – *No* and 1 – *Yes*. In order to avoid scaling bias, responses to item 3 were dichotomized into the absence (score of 0 or 1) or presence of problematic complaints (scores of 2 or 3). We opted for this method as suggested by the original author (Schmand et al., 1996) to differentiate clinically relevant complaints.

### Outcome Definitions

Language outcome was evaluated with the *z*-scores obtained in the following two language tests at follow-up: the 1-min trials semantic and phonemic fluency. In addition, individuals were classified at baseline as having “*Language Complaints*” (LC) if both SMCQ items had a score of 1 or “*No Language Complaints*” (NLC) if a score of 0 was obtained in at least one of those items (NLC).

The diagnosis of cognitive impairment (MCI/dementia) was done in accordance to published criteria (APA, 1994; Petersen, 2004) and has already been described in previous work (Martins et al., 2018). Briefly, it was obtained by consensus and involved information from the results obtained in memory and executive tests contributing to the composite indexes, the review of all available clinical, neuropsychological, and imaging data by a panel of two neurologists and two neuropsychologists, and an independent clinical evaluation performed in a research center (not presented in study data) with a minimum interval of 6 months after study testing.

### Data Analysis

Data were analyzed using IBM SPSS computer software (SPSS Inc., Chicago, IL). Descriptive statistics was used for continuous variables and counts (percent) for categorical variables. Group differences were tested using the Student’s *t*-test for continuous variables or Chi-Square (*χ*^2^) test for categorical variables. To investigate whether the presence of language complaints at baseline was a predictor of poorer language performance at follow-up, multivariate linear regression analysis was used, with correction for sex, depressive symptoms, living status and baseline performance, executive and memory composite scores. Whenever statistical differences existed between groups on baseline measures, change between baseline and follow-up performances were used as an independent variable in the regression models. Due to the multiplicity of tests, Bonferroni correction was used and adjusted *p* for each comparison were computed by multiplying uncorrected *p* with the number of tests conducted in each analysis. Results were considered statistically significant at *p* < 0.05.

## Results

At follow-up, participants had a mean age of 70.4 (±8.3) years, 7.5 (±4.4) years of education, 63.6% were female, and 13.6% were living alone (Table 1). No statistical differences were found between LC and NLC groups with respect to these variables. At baseline, 47.6% of the subjects scored on SMCQ_Item 6_ and 31.7% on SMCQ_Item 3_. Altogether 25 participants were classified as LC. The LC group had significantly lower baseline performance on semantic verbal fluency (animals; *p* < 0.05) and had more depressive symptoms and overall cognitive complaints (*p* = 0.0014). At follow-up, LC group showed significantly lower scores on semantic (animals; *p* = 0.014) and phonemic fluencies (*p* = 0.0014).

**Table 1:** Baseline variables by language complaint group.

	Total (*n* = 275)	Language Complaints at baseline		Statistics^*^	Bonferroni-adjusted *p*
		LC	NLC		
Gender (M:F)	99:176	6:19	89:151	1.685	1.000
Age at follow-up [yrs; mean (SD)]	70.4 (8.3)	70.5 (8.7)	70.1 (8.2)	0.202	1.000
Education [yrs; mean (SD)]	7.5 (4.4)	8.3 (4.7)	7.1 (4.3)	1.430	1.000
Living status (alone:not alone)	37:235	5:20	30:210	1.111	1.000
**Baseline measures**					
MMSE [mean (SD)]	28.3 (1.8)	28.1 (2.0)	28.3 (1.7)	−0.679	1.000
Semantic fluency (food) [*z*-score; mean (SD)]	0.6 (1.6)	0.1 (1.4)	0.7 (1.6)	−2.459	0.196
Semantic fluency (animals) [*z*-score; mean (SD)]	0.2 (1.0)	**−0.3 (0.9)**	0.2 (01.0)	**−3.401**	**0.014**
Phonemic fluency [*z*-score; mean (SD)]	−0.2 (1.0)	−0.5 (1.0)	−0.2 (1.0)	−1.930	0.756
GDS [mean (SD)]	3.5 (3.4)	**5.6 (2.5)**	3.3 (3.3)	**4.215**	**0.0014**
SMCQ [mean (SD)]	6.1 (3.1)	**11.5 (2.5)**	5.6 (3.2)	**11.653**	**0.0014**
Vocabulary [mean (SD)]	52.1 (14.0)	53.0 (13.6)	52.1 (14.1)	0.424	1.000
**Follow-up measures**					
Semantic fluency (food) [*z*-score; mean (SD)]	0.7 (1.6)	0.1 (1.4)	0.8 (1.6)	−2.036	0.602
Semantic fluency (animals) [*z*-score; mean (SD)]	0.1 (1.0)	**−0.6 (0.9)**	0.2 (1.0)	**−3.411**	**0.014**
Phonemic fluency [*z*-score; mean (SD)]	−0.1 (1.0)	**−0.8 (1.1)**	−0.1 (1.0)	**−3.901**	**0.0014**

* *Student’s *t*-test (continuous variables) or *χ*^2^ test (categorical variables)*.

*F, female; GDS, Geriatric Depression Scale; LC, Language Complaints group; M, male; MMSE, Mini-Mental State Examination; NLC, No Language Complaints group; SMCQ, Subjective Memory Complaints Questionnaire; yrs, years; bold values represent statistical significance at the *p* < 0.05 level*.

**Figure 1:**
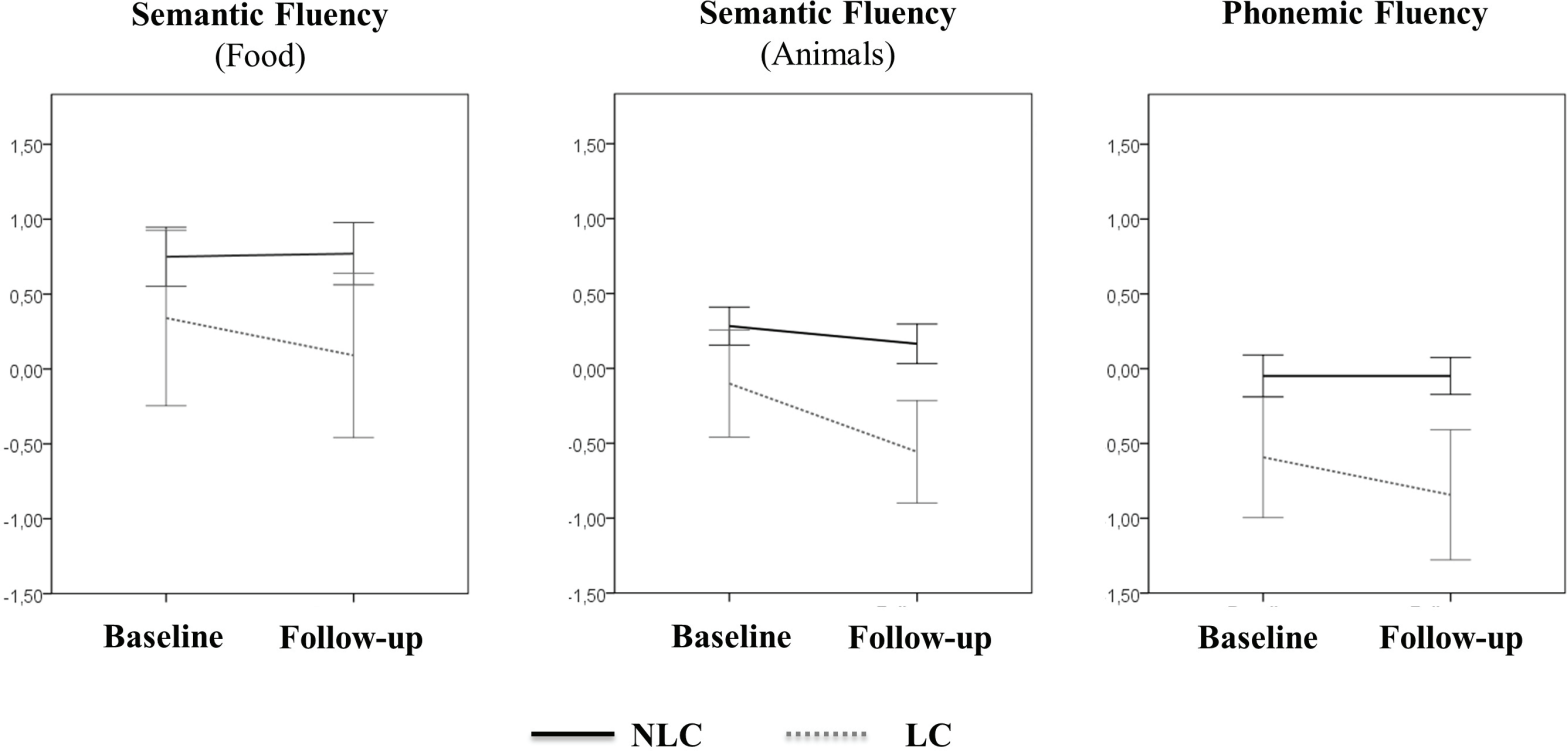
Longitudinal changes in verbal fluency tests’ scores. Figure 1 shows the longitudinal slopes for changes in verbal fluency at follow-up. Tests’ scores are presented as estimated marginal means after adjusting for age and years of education. Error bars represent standard error of the mean.

To investigate whether the presence of language complaints at baseline was a predictor of worse language performance at follow-up, a multivariate linear regression analysis was used, with correction for sex, depressive symptoms, living status, baseline performance, executive, and memory composite scores. Models for semantic (food and animals categories) and phonemic fluency were statistically significant [*F*(6,214) = 11.382, *p* < 0.0001; *F*(6,215) = 14.617, *p* < 0.0001; *F*(6,215) = 19.969, *p* < 0.0001, respectively]. Overall, baseline performance predicted follow-up performance (*p* < 0.01). After controlling for possible confounders (Table 2) and adjusting *p* using Bonferroni correction, results indicate that the presence of LC was a significant independent predictor of follow-up performance on semantic (animals) (*p* = 0.005). Analyses were repeated using change (between baseline and follow-up) in fluency scores as independent variables, although models were not statistically significant [semantic/food; *F*(6,216) = 1.314, *p* = 0.252; semantic/animals: *F*(6,216) = 1.201, *p* = 0.307; phonemic: *F*(6,216) = 0.538, *p* = 0.799]. A trend toward more pronounced decline from baseline to follow-up is depicted in Figure 1.

**Table 2:** Baseline predictors of follow-up language performance (multivariable linear regression analysis).

Variables	Adjusted *R*^2^	Beta	*t*	Bonferroni-adjusted *p*	95% CI	
					Inferior	Superior
Semantic fluency (food)	0.221					
LC vs. NLC		−0.142	−2.291	0.161	−1.393	−0.105
Gender		**0.201**	**2.875**	**0.028**	**0.208**	**1.114**
GDS		−0.051	−0.763	1.000	−0.092	0.041
Alone		0.004	0.073	1.000	−0.537	0.578
Baseline performance		0.158	2.503	0.091	0.036	0.304
Executive composite score		**0.239**	**3.728**	**0.0007**	**0.155**	**0.502**
Memory composite score		**0.179**	**2.708**	**0.049**	**0.083**	**0.530**
Semantic fluency (animals)	0.280					
LC vs. NLC		**−0.169**	**−2.826**	**0.035**	**−0.973**	**−0.173**
Gender		0.003	0.042	1.000	−0.271	0.283
GDS		0.054	0.840	1.000	−0.023	0.058
Alone		0.120	2.031	0.301	0.010	0.698
Baseline performance		**0.460**	**7.576**	**0.0007**	**0.355**	**0.605**
Executive composite score		0.098	1.603	0.777	−0.020	0.193
Memory composite score		0.041	0.631	1.000	−0.096	0.186
Phonemic fluency (P)	0.344					
LC vs. NLC		−0.124	−2.169	0.217	−0.781	−0.037
Gender		−0.054	−0.843	1.000	−0.366	0.147
GDS		−0.031	−0.500	1.000	−0.048	0.028
Alone		0.039	0.697	1.000	−0.206	0.431
Baseline performance		**0.522**	**9.025**	**0.00071**	**0.379**	**0.590**
Executive composite score		0.105	1.772	0.546	−0.010	0.189
Memory composite score		0.049	0.796	1.000	−0.077	0.181

*CI, confidence interval; GDS, Geriatric Depression Scale; LC, Language Complaints group; NLC, no language complaints group; bold values represent statistical significance at the *p* < 0.05 level*.

In addition, gender (*p* = 0.004), executive (p < 0.0001), and memory baseline performances (*p* = 0.007) were also significant predictors of semantic (food) fluency.

The presence of language complaints was not significantly associated with a clinical diagnosis of cognitive impairment (MCI or dementia) at follow-up (*χ*^2^ = 0.766, *p* = 0.381).

## Discussion

In this longitudinal study of a cohort of community-dwelling adults followed in primary care services, we could confirm the presence of an objective decline in language-based tests after a median of 5 years of follow-up when significant LCs were present at inception. Almost half of our sample reported difficulties finding particular words in conversation and, to a lesser extent, difficulties remembering family and friends’ names. A recent study aiming to explore specific items of a subjective cognitive decline questionnaire that discriminate pre-AD from normal aging reported similar frequencies. Valech et al. (2018) found that 46.2% of the controls scored on a similar item, while 44.2% reported difficulties in recalling famous and recently learned names. This highlights the relevance of LC in the context of broader SCC.

Word-finding is a complex process that requires access to the semantic memory system, subsequent search, and lexical retrieval of the phonological code (Indefrey and Levelt, 2004), which are dependent on an extensive network located in anterior and posterior regions of the temporal cortex of the left hemisphere (Gesierich et al., 2012). Word-finding problems for common and proper names are extremely frequent with the advancing age. The “*tip-of-the-tongue*” phenomenon usually reflects slowing and a less accurate lexical retrieval due to aging process (Mortensen et al., 2006) despite the fact that elderly subjects are able to maintain or improve their vocabulary and knowledge of word meaning (Salthouse, 2019). Such difficulties have been related to a poorer access to lexico-semantic operations and representations due to a slowdown in executive functions (Salthouse et al., 2003; Baciu et al., 2016) that arise from age-related changes in brain activation patterns of prefrontal regions (Cabeza et al., 1997; Dennis et al., 2008). This holds particularly true for proper naming whose retrieval process is usually perceived as more difficult than naming of common names.

Proper and common naming are known to dissociate at the lexical access level (Martins and Farrajota, 2007), and self-reported problems in both domains tend not to correlate with one another, when assessed by simple self-rating subjective cognitive complaints questionnaires (Martins et al., 2012a,b). In proper naming, the subjects have to access to a unique feature (the name) of a single entity in a highly specific process mediated by a frontotemporal network (that includes the temporal pole and inferior frontal gyrus), much more susceptible to the effects of the aging brain (Rotschtein et al., 2005; Pisoni et al., 2015).

The variety of processes involved indicate that subjective language complaints may correspond a variety of impairments, ranging from lexical/phonological search or retrieval problems to learning and storing difficulties. These loci of impairment may be associated with different pathologic processes during aging.

In the present study, we analyzed the effect of baseline LC as a risk factor for poorer performance or increased decline on language measured by verbal fluency tasks. From a cognitive standpoint, the tests applied involve language/semantic abilities (vocabulary and lexical/semantic retrieval) as well as high executive processing (e.g., working memory, processing speed, and self-monitoring) (Whiteside et al., 2016), thus tackling the functions associated with word retrieval. These differences become even more evident when we look into the distinct nature of semantic and phonemic fluency: the first relies upon semantic knowledge, whereas the second relies more upon executive functioning (Friesen et al., 2015). This led us to hypothesize that with time, phonemic fluency performances would be more sensitive to aging and suffer a more pronounced decline. Interestingly, in our sample, and after controlling for memory and executive performance, having LC was more predictive of poorer semantic fluency 5 years later. These results are corroborated by a recent study aiming to analyze the performance of semantic and phonemic fluency tasks in a sample of 86 controls with ages ranging from 30 to 89 years old (Gordon et al., 2018). The authors observed that semantic fluency depends largely on lexical retrieval speed and upon visualization strategies to support controlled retrieval skills which may disproportionately decline with age.

Although having language complaints at baseline was not significantly predictive of a higher decline nor the diagnosis of MCI/dementia 5 years later, these individuals showed worse objective performances at this time corroborating that their complaints had an objective translation. Future studies should address whether this single domain is declining at a faster pace than global cognitive decline in subjects presenting with significant language complaints.

In line with previous work from our group (Martins et al., 2012a,b), living alone was a significant predictor of follow-up semantic fluency performance. Living status has been reported in several cognitive aging studies as an important variable. In a previous study from our group, subjective language complaints were more common in subjects living alone (Martins et al., 2012a). This might eventually suggest that maintaining regular conversations decreases language difficulties, which is in agreement with other findings about cognitive performance. Indeed, social interactions can be viewed as natural forms of cognitive stimulation and may play a relevant part on the stimulation of language skills, whereas living alone would represent a situation of relative cognitive deprivation, with reduced cognitive stimulation and lower cognitive reserve (e.g., processing speed) (Bennett et al., 2006; Gow et al., 2013). Being male was also a predictor of poorer performance of semantic (food) fluency. Previous studies have shown that women tend to outperform men in verbal fluency particularly in specific categories such as “food/supermarket”/“fruits” categories (Capitani et al., 1999; Santos Nogueira et al., 2016), which may indicate that different semantic categories have distinct representation in the cognitive system.

We acknowledge some limitations to this study. The first is the high attrition rate. As previously reported (Martins et al., 2018), individuals that were lost to follow-up were older and had lower baseline MMSE than those who remained in the study. The results presented may therefore underscore the risk of a 5-year decline. Secondly, we used a rather conservative classification of cognitive complaints, requiring that subjects had complaints in both language questions. This resulted in a small number of affected individuals for comparison which reduced statistical power leading to non-significant differences between groups in decline overtime when using change in verbal fluency performance as an independent variable (despite the suggestive trend toward a faster decline in the LC as depicted in Figure 1). Thirdly, we cannot exclude the presence of subjects with cognitive impairment at baseline, as an explanation for their lower objective performance at that timepoint. The fact that these individuals did not differ from controls on the vocabulary test at baseline, a test that is usually resistant to aging, suggests that their impairment was not due to a developmental process. In this case, their persistent language complaints indicate an ongoing acquired process that translates into worse performance on language-based measures. However, the current total time (5 years) might be short to detect significant changes between groups overtime. Finally, generalization of the present findings is limited since it cannot be guaranteed that the poorer performance of the LC group is related at some extent to the effect of a broad age-associated cognitive decline on performance. Future studies should address the predictive value of language complaints on a specific linguistic measure in order to ascertain the effect value of this type of complaints.

The 5-year follow-up, the use of a comprehensive neuropsychological at baseline, and the clinical data provided by general practitioners at the primary care centers were strengths of this study.

## Conclusion

Subjective language difficulties may represent a domain-specific cognitive complaint, that is associated to objects poor performance 5 years later in tests highly depending on language abilities, when compared to subjects not reporting such difficulties. Although more studies are necessary to disentangle the contribution of different cognitive processes to these complaints, and their long-term impact, they must be valued since they may allow the identification of subjects that have an objective worse performance in language tasks.

## Data Availability

The datasets for this manuscript are not publicly available because Datasets belong to Language Research Laboratory (Faculty of Medicine of Lisbon) and are currently still being analyzed. Requests to access the datasets should be directed to isabel_martins@medicina.ulisboa.pt.

## Author Contributions

Initial concepts and framework were developed by CM and IM. Data analysis and interpretation were performed by CM and IM. Manuscript was prepared by CM. IM helped with revision and final comments.

### Conflict of Interest Statement

The authors declare that the research was conducted in the absence of any commercial or financial relationships that could be construed as a potential conflict of interest.
